# Anterior Ethmoidal Artery Evaluation on Coronal CT Scans

**DOI:** 10.1016/S1808-8694(15)30839-9

**Published:** 2015-10-18

**Authors:** Soraia Ale Souza, Marcia Maria Ale de Souza, Luís Carlos Gregório, Sergio Ajzen

**Affiliations:** 1 Master’s degree in radiology, UNIFESP; graduate doctoral student, UNIFESP.; 2 Doctorate in otorhinolaryngology, UNIFESP; post-doctoral student, UNIFESP.; 3 Doctorate in otorhinolaryngology, UNIFESP; adjunct professor of the otorhinolaryngology discipline, UNIFESP.; 4 Livre docencia certification, UNIFESP; full professor of the Image Diagnosis Department, UNIFESP. Universidade Federal de Sao Paulo and Laboratório Diagnosticos da America.

**Keywords:** anatomy, ethmoid sinus, tomography

## Abstract

The anterior ethmoidal artery (AEA) is an important point for frontal and ethmoid sinuses surgery. CT scans can identify landmarks to help the surgeon find the AEA. **Aim:** To identify the landmarks of the AEA on the orbital medial wall and on the lateral wall of the olfactory fossa. and to correlate the presence of supraorbital ethmoidal cells with spotting the anterior ethmoidal artery canal. **Materials and Methods:** Retrospective review of 198 direct coronal paranasal sinuses computed tomography (CT) scans from August to December, 2006. **Results:** Supraorbital pneumatization was seen in 35% (70 scans). The AEA canal was seen in 41% (81 scans). The anterior ethmoidal sulcus was seen in 98% (194 scans) and the anterior ethmoidal foramen was seen in all the scans (100%). **Conclusion:** The anterior ethmoidal foramen and the anterior ethmoidal sulcus were anatomical landmarks present in almost 100% of the scans studied. There was a correlation between the presence of supraorbital pneumatization and AEA canal visualization.

## INTRODUCTION

The anterior ethmoidal artery crosses three cavities: the orbit, the ethmoid labyrinth and the anterior fossa of the skull. In enters the olfactory fossa through the lateral lamella of the cribiform plate along the so-called anterior ethmoidal sulcus, which is the point of greatest frailty of the whole anterior skull base. At this point the bone is extremely thin, and is considered as a high-risk area in nasal endoscopic surgery. In its course through the ethmoid labyrinth, the position of the anterior ethmoidal artery relative to the ethmoidal roof is very variable; the artery thus becomes vulnerable to injury during surgical procedures.^1^, ^2^

This artery irrigates the anterior ethmoidal cells and the frontal sinus; it also gives rise to the meningeal vessels in its course along the olfactory fossa, and also descends to the nasal fossa to irrigate the anterior thirds of the nasal septum and the lateral wall of the nose.^1^, ^3^, ^4^, ^5^, ^6^, ^7^, ^8^, ^9^

The anterior ethmoidal artery is an anatomical landmark; its location is important for recognizing structures of difficult access (frontal sinus) and to define the superior limits in surgery (skull base).^10^, ^11^, ^12^, ^13^, ^14^ Additionally, visualizing this artery makes it possible to recognize and treat causes of severe epistaxis.^1^

Kainz and Stammmberger^15^ reported that the anterior ethmoidal canal may be in direct contact with the skull base, particularly when the roof of the ethmoid sinus is low. In most cases, however, a mesentery connects the canal to the roof of the ethmoid sinus, and there may be a space of up to 5 mm between the anterior ethmoidal artery and the roof. Becker,^16^ likewise, found a similar anatomical situation in endoscopic dissections. Moon et al.,^11^ however, contrary to these studies, found in anatomical specimens that the anterior ethmoidal artery coursed freely through the anterior ethmoidal cells in only 11% of cases, and that in made direct contact with the skull base in 85.7% of cases.

Gotwald et al.^17^ used a coronal tomographic analysis to investigate anatomical landmarks for locating the anterior ethmoidal artery, and found that the notch in the medial wall of the orbit (anterior ethmoidal foramen) and the focal funneling in the olfactory fossa (anterior ethmoidal groove) were good references for identifying the position and the orientation of the anterior ethmoidal artery within the ethmoid sinus.

Simmen et al.^13^ demonstrated in an endoscopic study that the position of the anterior ethmoidal artery varies significantly, and that the artery was located below the skull base in the presence of a pneumatized supraorbitary ethmoid sinus.

Accidental injuries of the lateral lamella of the cribiform plate and the anterior ethmoidal artery are the main potential risks during endoscopic surgery, and may cause disastrous consequences.^12^, ^18^, ^19^, ^20^, ^21^, ^22^, ^23^, ^24^, ^25^ Knowledge of the complex anatomy of the skull base and its anatomical landmarks, including the ethmoidal fovea, the lateral lamella of the cribiform plate, and the course of the anterior ethmoidal artery is essential to prevent the complications of nasal endoscopic surgery.^12^, ^15^, ^20^, ^21^, ^26^

Computed tomography^22^ (CT) has helped not only to evaluate nasosinusal disease, but also to characterize the anatomy of the paranasal sinuses.^22^, ^27^, ^28^, ^29^, ^30^ The coronal plane, in particular, is considered as a map for assessing the anatomy that varies significantly even between both sides in the same individual; this may alert about areas of potential complication risk in nasal endoscopic surgery.^18^, ^28^, ^30^, ^31^, ^32^, ^33^, ^34^, ^35^, ^36^, ^37^

## SERIES AND METHOD

A retrospective analysis was made of 198 computed tomographies of the paranasal sinuses done from August to December 2006 at the units of the Diagnosticos da America laboratory in the city of Sao Paulo.

Exclusion criteria were as follows: patients aged below 12 years, a history of surgery or trauma in the paranasal sinuses or the skull base, congenital anomalies of the face, paranasal sinus malignancies, osteofibrous lesions, and sinus diseases that opacified the frontal recess and/or the anterior ethmoidal cells.

The Research Ethics Committee of the institutions approved the project (number 0757/05).

### Computed Tomography

All of the exams were done using a Hi-Speed (GE Medical Systems, Milwaukee, USA) spiral CT unit. Only images in the coronal plane were used for study and analysis. These planes were made with patients in ventral decubitus, using perpendicular sections to the hard palate, from the anterior border of the frontal sinus to the anterior border of the clivus.

The technical imaging settings are shown below in Table 1.

**Table 1:** Technical parameters for gathering and analyzing the tomographic images.

Technical settings					
kV	mAs	Field of vision	Filter	Thickness/slice increment	Window (Width/Level)
120	150	14,7cm	Bone	3/3mm	2500/400 UH

### Analysis of the CT exam

**Figure 1:**
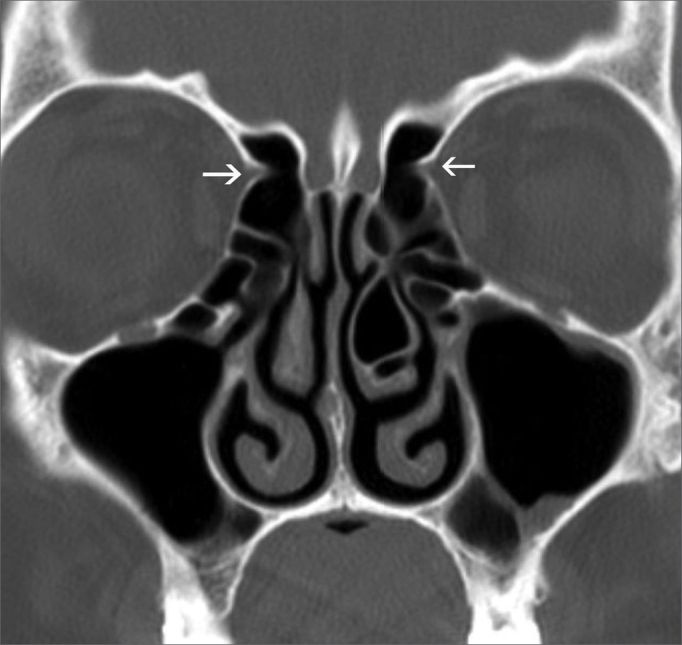
Anterior ethmoidal foramen - bony notch (arrows) on the medial wall of the orbits, corresponding to the anterior ethmoidal foramens.

**Figure 2:**
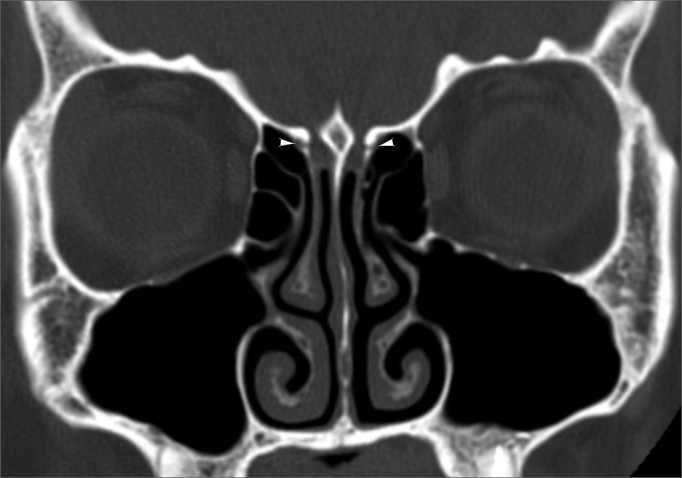
Anterior ethmoidal sulcus - bony sulcus (tip of arrows) on the lateral walls of the olfactory fossae, corresponding to the anterior ethmoidal sulci.

A single observer interpreted all exams based on the following parameters:

Anatomical landmarks used for identifying the anterior ethmoidal artery: the bony notch on the medial wall of the orbit, which corresponds to the anterior ethmoidal foramen (Figure 1), and the bone sulcus on the lateral wall of the olfactory fossa, which corresponds to the anterior ethmoidal sulcus (Figure 2).

**Figure 3:**
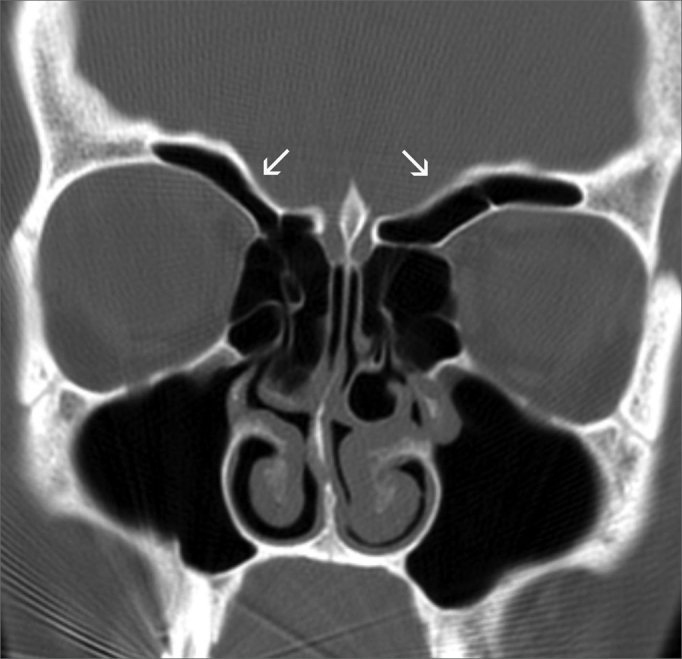
Supraorbitary pneumatization - ample bilateral supraorbitary pneumatization (arrows)

Presence of supraorbitary pneumatization, which Owen and Kuhn^38^ defined as roof of the orbit pneumatization postero-laterally to the frontal recess (Figure 3).

**Figure 4:**
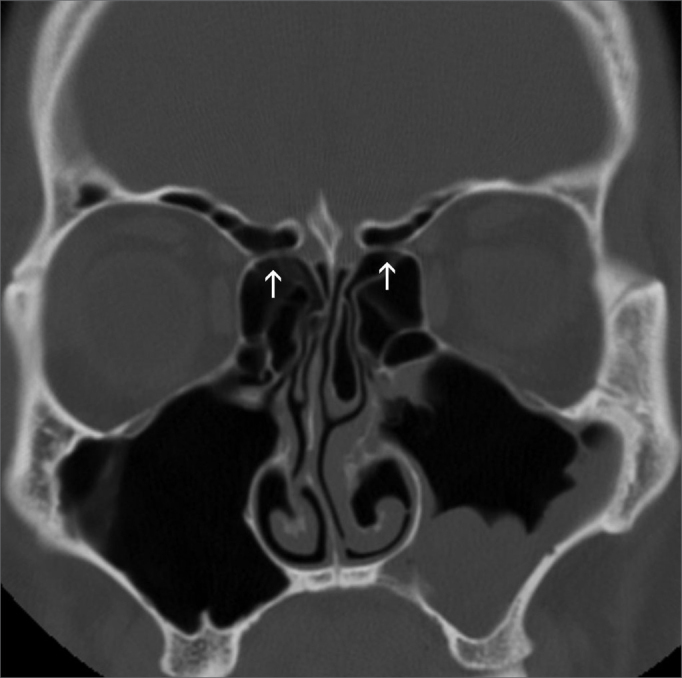
Anterior ethmoidal canal - anterior ethmoidal canal in its course through the anterior ethmoidal cells (arrows).

Anterior ethmoidal artery canal is seen, showing its course fully or partially (Figure 4).

### Statistical analysis

Data were stored in a database and analyzed using the SPSS 10 for Windows software. A descriptive analysis was made of the frequency distribution of qualitative variables. The chi-square test or Fisher’s Exact Test were applied as appropriate for comparing the prevalence of categorical variables. P values below or equal to 0.05 were defined as statistically significant.

## RESULTS

### Distribution of frequencies

**Figure 5:**
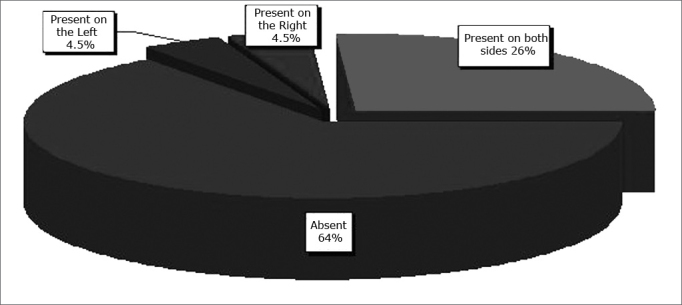
Supraorbitary pneumatization - distribution of supraorbitary pneumatization according to the side in which it was seen.

The sample consisted of 198 patients, 82 males (41.4%) and 117 females (58.6%). The age ranged from 12 to 88 years (mean - 34.33 years). Supraorbitary pneumatization was found in 35% of the exams; it was bilateral in 27% of exams (Figure 5).

**Figure 6:**
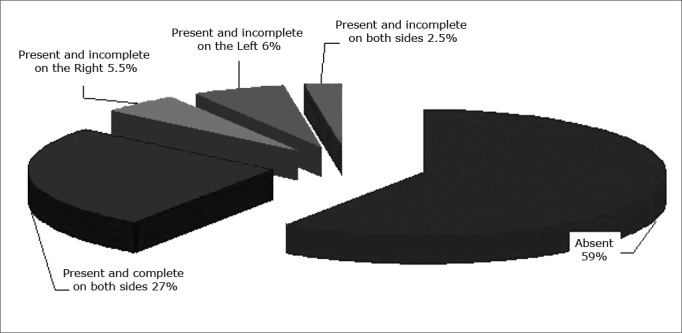
Anterior ethmoidal artery canal - distribution of visualization of the anterior ethmoidal artery canal according to the side and the type of characterization.

The anterior ethmoidal artery canal was seen in 41% of exams; most of them were seen completely (Figure 6).

The anterior ethmoidal sulcus was seen in 98% of exams; the notch on the medial wall of the orbit, which corresponds to the anterior ethmoidal foramen, was seen in 100% of exams.

### Association among the categories

Table 2 shows the statistically significant associations between the presence of supraorbitary pneumatization and visualization of the anterior ethmoidal artery canal.

**Table 2:** Distribution of the visualization of the anterior ethmoidal artery canal in relation to supraorbitary pneumatization

Visualization of the anterior ethmoidal artery canal	Pneumatization		
	Absent	Present	Total
Absent	118 (59,6%)	-	118 (59,6%)
Present	11 (5,6%)	69 (34,8%)	80 (40,4%)

Total	129 (65,2%)	69 (34,8%)	198 (100,0%)

Groups were statistically different (p<0.001).

## DISCUSSION

There is ample variation in the course of the anterior ethmoid canal in the ethmoid sinus.^4^, ^5^, ^12^, ^39^ Injury of the anterior ethmoidal artery during endoscopic procedures may occur, with severe consequences. Preoperative knowledge of the course of the artery is essential to avoid complications; this important task belongs to CT.

Many anatomical studies for locating the anterior ethmoidal artery have been published; most of them use endoscopic measurements related to endonasal anatomical landmarks.^1^, ^3^, ^4^, ^5^, ^8^, ^9^, ^11^, ^12^, ^13^, ^14^, ^40^, ^41^ On the other hand, we found few papers describing studies using CT.^6^, ^17^, ^18^, ^33^, ^39^

In assessing the anatomical landmarks for locating the anterior ethmoidal artery, we found that the medial notch of the orbit (anterior ethmoidal foramen) and the anterior ethmoidal sulcus on the lateral wall of the olfactory fossae were reliable parameters, since they were seen respectively in 100% and 98% of CT exams. Gotwald et al.^17^ assessed 40 coronal plane CT exams using similar technical settings as ours, and showed that the medial notch of the orbit and the anterior ethmoidal artery sulcus were found respectively in 95% and 84% of exams. McDonald et al.^7^ analyzed 50 CT exams in the coronal plane with 2.5 mm contiguous slices, and found the anterior ethmoidal foramen bilaterally in 95% of exams, and unilaterally in 5% of exams; these results were similar to ours. These authors did not, however, assess the presence of the anterior ethmoidal foramen, as was done in our study.

We characterized the anterior ethmoidal artery canal in its course within the ethmoid sinus in 41% of exams. This result was similar to those of Basak et al.,^18^ who studied the location of the anterior ethmoidal artery relative to the skull base using coronal plane CT, and characterized the artery in its course through the anterior ethmoid cells in 43% of exams. Gotwald et al.^17^ identified the probable orientation of the course of the anterior ethmoidal artery in 79% of their CT exams, but used only anatomical landmarks without directly visualizing the anterior ethmoidal artery canal within the ethmoid sinus.

Supraorbitary pneumatization was seen in 35% of exams. This rate is somewhat higher than that of Chung et al.,^42^ who also used coronal plane CT with 3 mm thickness slices to find a 26% rate in their sample. These authors, differently from us in our study, did not investigate any association between this occurrence and identifying the course of the anterior ethmoidal artery.

There was a statistically significant association between the presence of supraorbitary pneumatization and visualization of the anterior ethmoidal artery canal. The artery canal was seen in all exams in which supraorbitary pneumatization was present. We found that the presence of supraorbitary pneumatization influenced the relation between the artery and the skull base. We found in all cases that the artery coursed freely through the ethmoidal cells, positioned far from the ethmoidal roof. In a CT study, Bayram et al.^33^ alerted about this possible association, but did not assess the frequency of this finding. Similarly, Simmen et al.^13^ also found the anterior ethmoidal artery canal below the ethmoidal roof in the presence of supraorbitary pneumatization in anatomical dissections of 30 half skulls. Thus, preoperative recognition of supraorbitary pneumatization in CT is valuable, as it provides an alert that the anterior ethmoidal artery canal is far from the ethmoidal roof, and courses freely within the ethmoidal cells; this increases significantly the risk of injury of the anterior ethmoidal artery during a surgical procedure.

## CONCLUSION

The medial notch of the orbit (anterior ethmoidal foramen) and the anterior ethmoidal sulcus on the lateral wall of the olfactory fossae were reliable parameters for identifying the course of the anterior ethmoidal artery; they were characterized in almost 100% of exams.

The course of the anterior ethmoidal artery was identified below the ethmoidal sinus roof in all exams in which supraorbitary pneumatization was present.
